# Virtual education and clinical practices in final-year Peruvian dentistry students during COVID-19 pandemic

**DOI:** 10.3389/fpsyg.2025.1468949

**Published:** 2025-04-10

**Authors:** Fiorella del Pilar Cabrera-Tasayco, Martín Andrés Chávez-Méndez, Javier Flores-Fraile, Claudio Peña-Soto, Myriam Angélica De la Garza-Ramos, Guillermo Cano-Verdugo

**Affiliations:** ^1^Universidad Científica del Sur, Facultad de Ciencias de la Vida y Salud, Carrera de Estomatología, Lima, Peru; ^2^Universidad de Salamanca, Departamento de Cirugía y Odontoestomatología, Salamanca, Spain; ^3^Universidad Autónoma de Nuevo León, Facultad de Odontología, Monterrey, Nuevo León, Mexico

**Keywords:** education, dental, online, students, clinical competence, COVID-19 pandemic, Peru

## Abstract

**Introduction:**

The COVID-19 pandemic has significantly altered various sectors, with education being one of the most impacted. In Peru, the shift from in-person to virtual education was imperative due to the pandemic’s constraints. This study investigates the perception of virtual education and clinical practice among final-year dentistry students at a Peruvian institution during the SARS-CoV-2 pandemic.

**Methods:**

Utilizing a cross-sectional design, data were collected from 97 students via a survey assessing their experiences and expectations related to virtual learning and clinical skills. Instruments with dichotomous response scales measured students’ experiences with virtual education and their expectations regarding clinical practice.

**Results:**

Results indicated that most students reported a moderate experience with virtual education, with similar moderate expectations for clinical practice. Statistical analyses revealed significant differences among experience levels and practice expectations, highlighting a disparity between students’ perceived skills and their confidence in applying these skills (*p* < 0.001).

**Discussion:**

The study’s findings suggest that while virtual education has provided continuity, there remains considerable room for improvement in both the quality of online instruction and practical training. Enhancements in virtual teaching methods and additional support for clinical practice could better meet the needs of dental students. This study emphasizes the importance of ongoing evaluation and adaptation of virtual education strategies to address the evolving challenges and improve educational outcomes for future dental professionals.

## 1 Introduction

The advent of SARS-CoV-2, also known as COVID-19, has profoundly impacted global daily life, leading to the cessation of activities in all countries to contain the spread of the virus. These measures included social distancing and widespread remote work across various sectors (Hattar et al., 2021; Padilla Machaca et al., 2020; Yadav et al., 2023).

One of the most affected sectors by the pandemic has been higher education. In Peru, the National Superintendency of Higher University Education through the Ministry of Education of Peru suspended in-person classes on March 27th, 2020, in response to the rising COVID-19 cases, necessitating a rapid transition to virtual education, a learning modality that utilizes digital platforms and online resources to deliver instructional content, enabling remote access to educational programs without the need for physical presence in a traditional classroom. Although this approach already existed, the pandemic made it the only viable alternative, facilitated by advances in information technology, thereby ensuring the continuity of education (Rios-Blancas et al., 2023; Sarwar et al., 2020; Ramanarayanan et al., 2022).

Virtual education has enhanced the efficiency of educators but has also posed the challenge of understanding how students perceive and respond to it. This transition has been particularly challenging for health science fields, such as dentistry, which require a combination of theoretical knowledge and practical skills, such as proficiency in performing clinical procedures, handling dental instruments, diagnosing oral conditions, and providing patient care in real-world settings (Ertürk Avunduk and Delikan, 2021; Bahanan et al., 2022). While virtual education can enhance pedagogical communication, universities face the difficulty of effectively integrating the theoretical and clinical components of dental education in a virtual environment. Nevertheless, the heavy load of theoretical classes has temporarily allowed these activities to adapt to virtual learning modalities, keeping students engaged and helping them progress in their careers (Klaassen et al., 2021). Specifically, in Peru, virtual education during COVID-19 took place through digital platforms, allowing students to interact with content and instructors online without being physically present. The most commonly used platforms in Peru for virtual education were Zoom and Microsoft Teams, with Microsoft Teams being particularly popular for course management and assessments (Schlenz et al., 2020; Ortega et al., 2020; Bączek et al., 2021; Badovinac et al., 2021; Keser and Namdar Pekiner, 2022).

Students’ ability to understand, communicate, and solve problems is essential for their performance in clinical training, which has been impacted by virtual education—particularly for final-year students in Peru, whose training is heavily focused on clinical practice. Previous studies have demonstrated that while virtual education is effective for theoretical courses, it cannot fully replace the hands-on practice required for professional dental training (Almhdawi et al., 2021; Herr et al., 2021; Antoniadou et al., 2022; Saxena et al., 2022). A study conducted in Greece found that dental students in Athens and Copenhagen considered online learning suitable only for theoretical instruction. However, they preferred in-person or hybrid education due to the lack of interaction and insufficient practical training (Antoniadou et al., 2022). In South Korea, research indicated that dental students adapted well to online pediatric dentistry education during the COVID-19 pandemic, expressing overall satisfaction and viewing university implementation as appropriate (Herr et al., 2021). Meanwhile, in India, a study revealed a significant correlation between practice level regarding online education among dental professionals, highlighting the need to enhance these aspects (Almhdawi et al., 2021; Saxena et al., 2022).

As we have seen, there are studies in other countries examining the impact of virtual education on health sciences. However, research specifically addressing its effects on clinical training in dentistry remains scarce, particularly in Latin America. Most available studies focus on theoretical learning, leaving a gap in understanding how the transition to virtual education has affected the acquisition of hands-on skills essential for professional competence. In developing countries like Peru, where access to digital resources and institutional preparedness may vary significantly, it is crucial to assess how these challenges influence clinical training and student perceptions.

Given the above, this study aims to analyze the perception of virtual education and clinical practices among final-year dentistry students at a university in the Metropolitan Area of Lima, Peru during the COVID-19 pandemic, addressing gaps in the literature regarding the specific impact on clinical training. We selected this university due to its strong emphasis on practical training in dentistry, and given the evolving landscape of virtual education, it is crucial to understand how these formats impact the acquisition of laboratory skills in dental training. In particular, we chose dental students from this university, because it is important to study how their learning was affected during COVID-19, as dental programs are highly practice-oriented and were subject to significant changes during this period. These shifts in learning environments potentially impacted their clinical skills development, which is essential for their future professional practice.

The research will answer key questions such as: How do final-year dentistry students perceive the effectiveness of virtual education for theoretical and clinical components during the COVID-19 pandemic? What are the expectations of final-year dentistry students regarding virtual clinical practice during the pandemic? How has the transition to virtual education impacted students’ confidence in applying their clinical skills? Based on these inquiries, the study hypothesizes that: (1) there is a significant difference in the perception of virtual education between theoretical and clinical components among final-year dentistry students, (2) final-year dentistry students perceive virtual clinical practice as less effective than in-person clinical practice, and (3) students’ expectations regarding virtual clinical education are moderate, reflecting a gap in the transition from traditional to virtual formats.

## 2 Materials and methods

We utilized a cross-sectional design for this study. The population consisted of dentistry students from a university in the Metropolitan Area of Lima, Peru. We selected the sample using a census-based method, which included all final-year students. This sample was based on non-probabilistic convenience sampling, as we selected 90% of the study population (*N* = 97), which did not require a formula due to the small size of the population. We included final-year dentistry students as the selection criterion and excluded those who had not taken theoretical and practical virtual classes or those who did not sign or did not wish to respond to the questionnaire. We eliminated incomplete records.

To measure the experience of virtual education and clinical practice expectations, the authors of this manuscript developed and validated two instruments based on the topics from Hattar et al. (2021). The first instrument, “Experience in Virtual Education for Dentistry Students,” aimed to assess students’ adaptation to and perception of virtual learning. It was designed through a multi-step process that included item selection, expert validation, and a pilot study. Initially, we identified key themes from the literature and previous studies on virtual education in dentistry. Based on these themes, we formulated nine items with dichotomous response scales (yes/no), with questions related to perceptions of virtual learning, clinical training, and student collaboration during the COVID-19 pandemic in a single dimension, ensuring clarity and relevance. Each positive response was weighted with two points and each negative response with one point. To ensure content validity, a panel of five experts in dental education and e-learning assessed the instrument, evaluating each item for clarity, coherence, and relevance. We conducted two rounds of revisions based on their feedback to refine ambiguous or redundant items. Following this, we conducted a preliminary pilot test with 15 students who were not part of the final sample. This test helped us assess item comprehension and response patterns, leading to minor modifications before full implementation. Finally, we categorized the total score of all questions as follows: 9 to 12 points, poor; 13 to 15 points, average; and 16 to 18 points, good experience in virtual education.

The second instrument, “Clinical Practice Expectations for Dentistry Students,” aimed to measure students’ self-perceived readiness for clinical practice and followed a similar validation process. We identified 18 key items aligned with two dimensions with questions related to perceptions of virtual learning’s impact on clinical skills, practical knowledge, professional development, and patient communication during the COVID-19 pandemic. The response scale was dichotomous (yes/no), with each positive response weighted with two points and each negative response with one point. Expert validation was conducted by a panel of dental educators, who provided structured feedback on item relevance and clarity. After incorporating their suggestions, we carried out a pilot study with 15 students to assess item interpretation and ensure consistency. Further refinements were made based on student feedback, improving item precision and eliminating potential misinterpretations. We categorized the total score of all questions as follows: 18 to 24 points, low; 25 to 30 points, medium; and 31 to 36 points, high practice expectations. Both instruments demonstrated good internal consistency, with Cronbach’s alpha values of 0.807 and 0.790, respectively, confirming their reliability (Bujang et al., 2018).

We conducted data collection in March, 2023, the date on which the National Superintendency of Higher University Education through the Ministry of Education of Peru authorized the return to in-person classes, and was considered as a period when students had just returned to in-person classes after a prolonged period of virtual learning. This timing was chosen as it provided an ideal moment to assess the transition from virtual education to in-person clinical practice, allowing for a clear comparison between their virtual experiences and the newly reintroduced face-to-face training. Data collection was via an email link to a Forms containing a general explanation of the study and its scope, along with the instrument to be applied. We gave participants a 72-h deadline to complete the survey, and sent a thank-you message upon submission.

For statistical analysis, we coded and analyzed the data using SPSS v25. Following normality tests, we applied the chi-square test to evaluate the difference between study variable groups. Values equal to or less than 0.05 were considered significant. Additionally, we conducted *post hoc* analyses based on standardized residuals to determine which specific groups contributed significantly to the observed difference in both variables.

This study received approval from the Institutional Ethics Committee of the Universidad Científica del Sur, under the registration code POS-53-2022-00059. To facilitate access to the student lists by cycle and academic year, we explained the issue and the purpose of the research in advance to the relevant authorities. Subsequently, we drafted the informed consent, detailing the study’s objective, the survey format, the voluntary participation of the students, and the confidentiality of personal data, in accordance with Law 27,933 of the Peruvian government. We processed the necessary requests through the university’s institutional email.

## 3 Results

Data collection involved 97 students. Detailed information regarding the sex and age of the participants appears in Table 1. Regarding the reported level of experience, nearly half of the respondents reported a moderate level of experience with virtual education. Statistical analysis revealed significant differences among the three reported experience levels (*p* < 0.001), with the “poor” level showing a significant difference compared to the other categories (*p* < 0.001) (Table 2). To further assess the magnitude of these differences, we calculated effect sizes using Cramér’s V, which indicated a moderate effect (*V* = 0.31) between experience levels. We also calculated confidence intervals for the proportions of experience categories, showing a 95% confidence interval of 0.09 to 0.21 for the difference between the ‘poor’ and ‘good’ experience levels.

**Table 1:** Demographic profile of the population.

Variable	*n*	%
Gender		
Male	26	26.80
Female	71	73.20
Age range		
18–20	22	22.68
21–23	37	38.14
24 and older	38	39.18

*N = 97. Direct survey.*

**Table 2:** Experience with virtual education among participants.

Experience level	*n*	%	*X* ^2^	*p* value
Good	44	45.36	32.30	< 0.001
Regular	47	48.45		
Poor	6	6.19*		

*N* = 97. *X*^2^ = Chi-square test. * = Significant value in post hoc tests. Direct survey.

**Figure 1:**
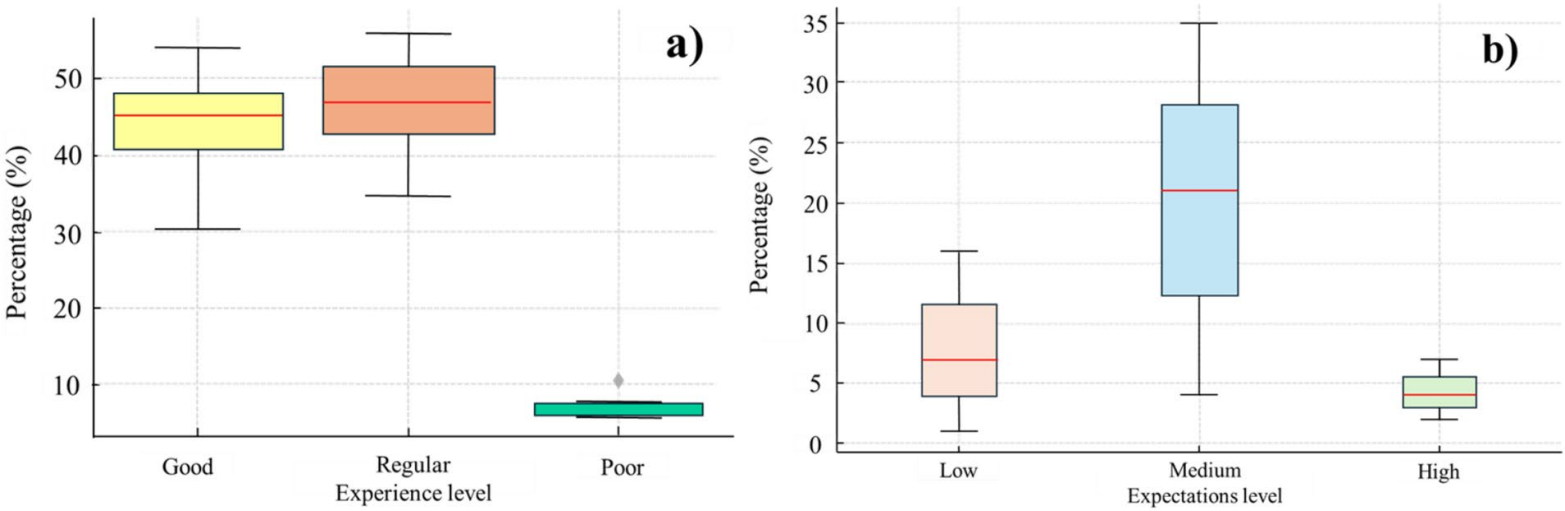
Graphical representation of main results. **(a)** Experiences in virtual education by experience level, **(b)** Practice expectations in virtual education by experience level.

Regarding the chi-square test results, *post hoc* analyses revealed that the “poor” experience level differed significantly from the “good” and “regular” categories, with a chi-square value of 32.30 (*p* < 0.001), indicating a notable discrepancy in the distribution of responses. The effect size of this difference, calculated using Cramér’s V, was moderate (*V* = 0.31), confirming that the differences in experience levels hold practical significance beyond statistical significance. This suggests that those with poor experiences may have faced unique challenges that were not as prevalent in the other groups (Figure 1).

Regarding participants’ clinical practice expectations, the overall analysis indicated significant differences among the three levels (high, medium, and low) (*p* < 0.001). Effect sizes for the chi-square test revealed a large effect (*V* = 0.40), indicating substantial differences in how students perceived their clinical practice expectations. The “medium” categories in both variables showed differences compared to the other categories, suggesting a notable disparity in the perception of acquired skills and confidence in applying these skills among the studied groups.

When analyzed by dimensions, it was reported that in the dimension of acquired clinical practice skills within the Dentistry program at a university, most participants consider their skills to be at a medium level. A smaller number perceive them as high, while a small proportion view them as low. A significant difference was reported among the three levels (*p* < 0.001), with the low level showing a statistical difference (*p* < 0.001). The confidence interval for the low level of acquired skills was 95% CI [0.20, 0.39], indicating that the difference is statistically robust and not likely due to random variation. Similarly, the dimension of confidence in applying these clinical skills also showed a significant difference among the three evaluated levels (*p* < 0.001), with the high level presenting a significant difference in the *post hoc* analysis (*p* = 0.04). The effect size for the difference between the high and medium categories was large (*V* = 0.45), and the confidence interval for the high level was 95% CI [0.12, 0.27], further supporting the practical significance of these findings (Table 3).

**Table 3:** Practice expectations among participants by instrument dimension.

Expectation level	*n*	%	*X* ^2^	*p* value
Acquired skills				
High	34	35.05	29.53	< 0.001
Medium	53	54.64		
Low	10	10.31*		
Confidence in skill application				
High	10	10.31*	34.41	< 0.001
Medium	57	58.76		
Low	30	30.93		

*N* = 97. *X*^2^ = Chi-square test. * = Significant value in post hoc tests. Direct survey.

The chi-square test indicates a significant relationship between practice expectations and the level of experience. However, the post hoc analysis did not identify specific significant differences among individual cells (*p* = 0.062), as the observed differences are likely due to the overall trend across groups. This finding suggests that while there is a general association between experience levels and practice expectations, the effect might be generalized rather than strongly pronounced in specific categories (Table 4).

**Table 4:** Practice expectations in virtual education by experience level.

	Expectations level				
Experience level (%)	Low (%)	Medium (%)	High (%)	*X* ^2^	*p* value
Good	16 (16.5)	21 (21.6)	7 (7.2)	9.52	0.049
Regular	7 (7.2)	35 (36.1)	4 (4.1)		
Poor	1 (1)	4 (4.1)	2 (2.1)		

*N = 97. X2 = Chi-square test. Direct survey.*

## 4 Discussion

The purpose of this study was to analyze the perception of virtual education and clinical practices among final-year dentistry students from a university in central Peru during the COVID-19 pandemic. The main results reported that the virtual education experience was moderate for most participants, as were their practice expectations.

When comparing these results with previous research, the findings show mixed patterns. For instance, this aligns with the observations made by Loch et al. (2021), who identified weaknesses and opportunities for improvement in virtual education. However, the results stand in contrast with those of Herr et al. (2021), who emphasized the positive effectiveness of virtual learning and the comfort students felt with it. Additionally, authors like Bączek et al. (2021), Schlenz et al. (2020), Badovinac et al. (2021), and Keser and Namdar Pekiner, (2022) conclude that dentistry students are also in favor of this type of education, which is in stark contrast to our findings. This discrepancy highlights a critical difference in the student body and university contexts.

The comparison of perceived preparation levels among dentistry students in this study indicates lower expectations than those reported by Abbasi et al. (2020), where 77% of students expressed negative perceptions about virtual education.

Concerning practical expectations, our results align with those of Antoniadou et al. (2022), who identified practical expectations as a challenge for virtual education. Similarly, Saxena et al. (2022) demonstrated that participants’ attitudes and practices were statistically significant. This finding aligns with the present study, which identified a significant relationship between virtual education and practice expectations.

From a theoretical perspective, these findings align with Kolb’s (1984), experiential learning theory, which emphasizes the importance of direct experience in the learning process. The limitations of virtual education in dentistry, particularly regarding clinical skill acquisition, challenge the effectiveness of online learning when applied to highly practical disciplines. Additionally, Vygotsky’s (1978), sociocultural theory suggests that interaction plays a crucial role in knowledge construction. The lack of hands-on experience and direct mentorship in virtual settings could explain the moderate perceptions reported in this study. These theoretical frameworks provide insight into why students may struggle to fully embrace virtual education in the context of clinical training.

This study’s main strength is its specific focus on how final-year dentistry students perceive virtual education and clinical practice during the COVID-19 pandemic. This focus provides a detailed and contextualized understanding of their experiences and expectations at a crucial stage in their professional training. Additionally, the study selects a representative sample from a university in the Metropolitan Area of Lima, Peru, to offer valuable insights into the local impact of virtual education during the pandemic. The research team chose this university for its strong emphasis on practical dental education, aligning with the study’s objectives. By comparing these results with previous national and international studies, the study strengthens the validity and relevance of its findings. This comparison deepens the analysis by identifying both similarities and differences in how students perceived virtual education during the crisis.

Studies on virtual education in practical dentistry classes present mixed perspectives. While researchers recognize the benefits of flexibility and increased access, they also highlight challenges related to direct hands-on interaction and equity in access to technology. Educators and institutions can overcome these barriers by integrating advanced technologies and developing hybrid methods to improve dental students’ education.

However, this study has some limitations that must be considered. First, the cross-sectional nature of the research limits the ability to draw causal conclusions or analyze changes over time. A longitudinal approach would provide more insights into how students’ perceptions evolve as they gain more experience with virtual learning. Additionally, the reliance on convenience sampling may introduce selection bias, potentially overrepresenting certain viewpoints while underrepresenting others. Another limitation is the self-reported nature of the data, which may be influenced by recall bias or social desirability bias. One limitation of the statistical analysis employed is the potential sensitivity of the chi-square test to small sample sizes and uneven group distributions. The unequal distribution of participants across experience categories may influence the robustness of the test, potentially affecting the generalizability of the results. Future studies should consider using alternative statistical methods, such as Fisher’s exact test or stratified sampling, to mitigate these issues and provide a more accurate analysis of the data.

The results of this study provide valuable insights for improving virtual education programs in dentistry. Since students perceive virtual education as ‘moderate,’ universities have a significant opportunity to enhance its quality and effectiveness. Institutions can use these findings to create strategies that strengthen online interaction, increase access to virtual clinical resources, and offer continuous support to students. For example, they can introduce clinical practice simulators, develop collaborative learning platforms, and conduct frequent feedback sessions to address the areas of dissatisfaction identified in the study.

Furthermore, the data underscore the significance of considering students’ expectations, as this can guide the realignment of educational programs to better meet the actual needs of the student body. By addressing these expectations, educational institutions can tailor their programs to more effectively respond to students’ demands, ultimately enhancing the learning experience. Implementing initiatives and fostering a more interactive, participatory virtual environment can enhance the online learning experience. Additionally, integrating advanced technological tools and ensuring ongoing faculty training in online teaching methods can further improve the quality of virtual education, equipping students with the professional competencies required for their future clinical practice.

Future research should focus on longitudinal studies to track changes in perceptions over time and evaluate the long-term impact of virtual education on clinical competency. Additionally, qualitative approaches, such as focus groups or in-depth interviews to triangulate findings and provide a more comprehensive understanding of student experiences, which could provide richer insights into students’ specific concerns and expectations, complementing the quantitative findings of this study. Lastly, comparative studies between universities with different virtual education models could help identify best practices and optimize online training strategies for dental education. Also should be considered expanding the sample size.

## 5 Conclusion

The experience of virtual education and practice expectations among final-year Peruvian dentistry students during the pandemic is moderate. During the pandemic, virtual education became the primary mode of teaching for dentistry students in Peru. Despite efforts to adapt programs and content to the digital environment, the general perception of final-year students is that the educational experience has been mostly moderate. This evaluation reflects both the technical and pedagogical challenges that have emerged during this period. Ongoing monitoring of students is crucial to identify and address the difficulties encountered. More research is needed to clarify the issues and barriers that have affected the quality of learning and students’ confidence in applying their acquired skills. To enhance virtual dental education, it is recommended to integrate advanced interactive tools, such as virtual reality-based simulators, augmented reality applications, and haptic feedback systems, to improve hands-on training in a digital environment. Additionally, hybrid learning models that combine online theoretical instruction with supervised in-person clinical practice should be prioritized. These approaches can help bridge the gap between virtual education and practical skill development.

From a policy perspective, it is essential to establish institutional frameworks that ensure equitable access to digital resources, improve faculty training in online pedagogy, and provide continuous student support programs. Implementing structured feedback mechanisms can also enhance the adaptability of virtual learning methodologies. In terms of future research, longitudinal studies should be conducted to assess how students’ perceptions and competencies evolve over time with virtual and hybrid education models. Comparative studies across different regions of Peru and international educational systems would be valuable in identifying best practices and context-specific strategies. Furthermore, research on the effectiveness of emerging digital learning tools in enhancing clinical skills could provide evidence-based recommendations for curriculum development. These initiatives will contribute to a better understanding of the impact of virtual education on clinical training and the design of evidence-based strategies for its continuous improvement.

## Data Availability

The data analyzed in this study is subject to the following licenses/restrictions: Supplementary data is available upon request. Requests to access these datasets should be directed to mchavezme@cientifica.edu.pe.
